# Importance of cardiac imaging assessment of epicardial adipose tissue after a first episode of myocardial infarction

**DOI:** 10.3389/fcvm.2022.995367

**Published:** 2022-11-14

**Authors:** Fabián Islas, Eva Gutiérrez, Victoria Cachofeiro, Ernesto Martínez-Martínez, Gema Marín, Carmen Olmos, Irene Carrión, Sandra Gil, Patricia Mahía, Miguel Ángel Cobos, Alberto de Agustín, María Luaces

**Affiliations:** ^1^Instituto Cardiovascular, Hospital Clínico San Carlos, Instituto de Investigación Sanitaria del Hospital Clínico San Carlos (IdSSC), Madrid, Spain; ^2^Departamento de Fisiología, Facultad de Medicina, Instituto de Investigación Sanitaria Gregorio Marañón (IiSGM), Universidad Complutense de Madrid, Madrid, Spain; ^3^Ciber de Enfermedades Cardiovasculares (CIBERCV), Instituto de Salud Carlos III, Madrid, Spain

**Keywords:** epicardial adipose tissue, myocardial infarction, infarct size, cardiac magnetic resonance, echocardiography

## Abstract

**Background:**

Over the past years, information about the crosstalk between the epicardial adipose tissue (EAT) and the cardiovascular system has emerged. Notably, in the context of acute myocardial infarction (AMI), EAT might have a potential role in the pathophysiology of ventricular structural changes and function, and the clinical evolution of patients. This study aims to assess the impact of EAT on morpho-functional changes in the left ventricle (LV) and the outcome of patients after an AMI.

**Methods:**

We studied prospectively admitted patients to our hospital with a first episode of AMI. All patients underwent percutaneous coronary intervention (PCI) during admission. Transthoracic echocardiography (TTE) was performed within 24–48 h after PCI, as well as blood samples to assess levels of galectin-3 (Gal-3). Cardiac magnetic resonance (CMR) was performed 5–7 days after PCI. Clinical follow-up was performed at 1 and 5 years after MI.

**Results:**

Mean age of our cohort (*n* = 41) was 57.5 ± 10 years, and 38 (93%) were male. Nine patients had normal BMI, 15 had overweight (BMI 25–30), and 17 were obese (BMI > 30). Twenty three patients (56%) had ≥ 4 mm thickness of EAT measured with echo. In these patients, baseline left ventricular ejection fraction (LVEF) after AMI was significantly lower, as well as global longitudinal strain. EAT thickness ≥ 4 m patients presented larger infarct size, higher extracellular volume, and higher T1 times than patients with EAT < 4 mm. As for Gal-3, the median was 16.5 ng/mL [12.7–25.2]. At five-year follow-up 5 patients had major cardiac events, and all of them had EAT ≥ 4 mm.

**Conclusions:**

Patients with EAT >4 mm have worse LVEF and GLS, larger infarct size and longer T1 values after a MI, and higher levels of Gal-3. EAT >4 mm was an independent predictor of MACE at 5-year follow-up. EAT thickness is a feasible, noninvasive, low-cost parameter that might provide important information regarding the chronic inflammatory process in the myocardium after an infarction.

## Introduction

Acute myocardial infarction (AMI) is the ultimate consequence of coronary artery disease (CAD) (1). It is known that CAD is associated with increased amounts of epicardial adipose tissue (EAT) (2). This EAT is found between the myocardium and the visceral pericardium and it can directly regulate myocardial response after a MI; without the presence of a muscle fascia, there is the possibility that crosstalk between the EAT and the myocardium occurs, causing an increase in the levels of secretory products -chemokines and cytokines- from EAT. This has a potential association with myocardial fibrosis development, with the functional consequences it might have (2, 3). The pathogenesis of CAD includes multiple mechanisms, such as inflammation, oxidative damage, endothelial dysfunction, lipidic accumulation, and glucotoxicity; EAT seems to have a role in all of these processes (4).

EAT thickness is an indicator of visceral adiposity, regardless of body mass index; moreover, some studies have demonstrated that EAT thickness quantified with echocardiography is related to clinical outcomes in MI with ST-segment elevation (4–6). In addition, cardiac magnetic resonance (CMR) provides robust morphological and functional information, as well as tissue characterization of the myocardium in patients with AMI (7).

This study aims to assess EAT's impact on morpho-functional changes in the left ventricle (LV) and the outcome of patients after an AMI through a multi-modality imaging perspective.

## Materials and methods

**Figure 1:**
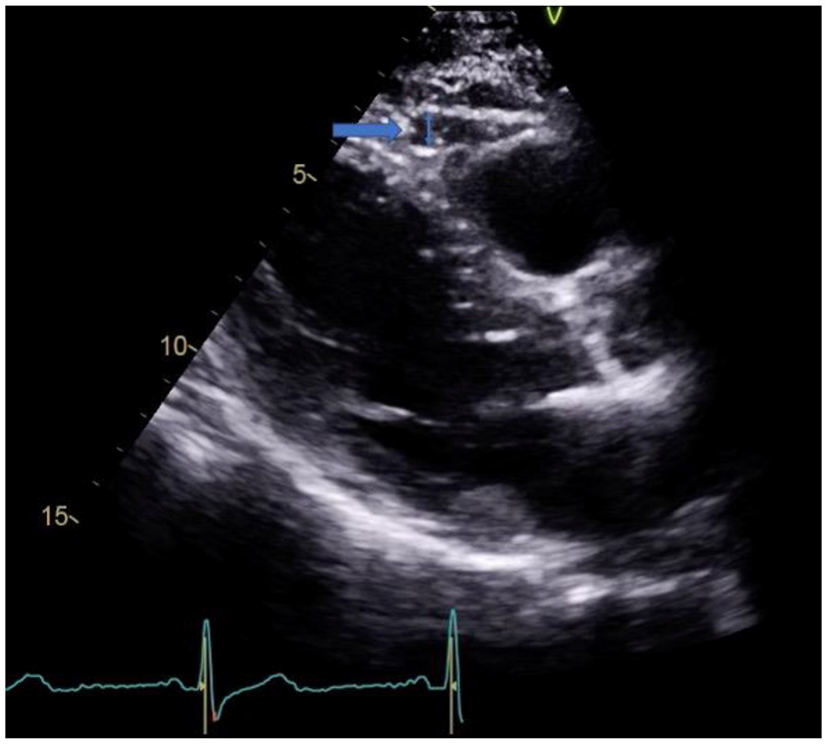
EAT in parasternal long axis view.

Single-center, observational, prospective cohort of 41 patients with a first episode of AMI treated with percutaneous coronary revascularization. All patients were admitted to the hospital as part of the protocol “Infarction Code” for primary percutaneous coronary intervention (PPCI). All patients underwent transthoracic echocardiography (TTE) within the first 24–48 h after hospital admission. The thickness of EAT was quantified in an end-diastole parasternal long-axis image at the level of the right ventricular free wall, Figure 1. 2D conventional parameters were obtained, as well as speckle tracking derived parameters, such as global longitudinal strain (GLS). CMR was performed 5–7 days after MI diagnosis with a 1.5 Tesla system and included steady-state free precession sequences (SSFP), and T1-weighted sequences for late gadolinium enhancement (LGE). LGE quantification was done by the semi-automated fullwidth at half-maximum (FWHM), defining infarct as myocardium with signal intensity (SI) > 50% of the peak SI in the infarct core. Microvascular obstruction (MVO) was defined as a hypo-enhanced region within the infracted myocardium in post-contrast images. T1 time quantification was obtained through Modified Look-Locker Inversion Recovery (MOLLI) sequences before and 15 min after the administration of gadolinium (0.2 mmol/kg). For myocardial extracellular volume (ECV), a region of interest (ROI) was placed in the LV myocardium to acquire pre- and post-contrast myocardial T1 values. At the level of the LV blood pool, another ROI was placed to assess the pre- and post-contrast blood T1 values. ECV was calculated with the following formula: ECV = (1 – hematocrit) Å~ (Δ R1 myocardium/Δ R1 blood). All images were analyzed with the software Medis Suite, version 3.2 (Medis Medical Imaging Systems. Leiden, The Netherlands).

Since our group of study has previously studied the effects of galectin-3 (Gal-3) on the cardiovascular system, and its role as a potential catalyst of myocardial damage by stimulating extracellular matrix deposition and amplification of pro-inflammatory mediators; we decided to evaluate levels of this marker in this cohort, at baseline, and during follow-up, to assess its potential association with EAT in the context of an AMI. The blood sample was collected in an ethylenediaminetetraacetic acid (EDTA) vacuum tube. The assay was performed and calibrated according to the manufacturer's protocol using an enzyme-linked immunosorbent assay.

Major adverse cardiovascular events during follow-up were defined as cardiovascular death, new myocardial infarction, admission for heart failure, new revascularization, or stroke.

### Statistical analysis

Continuous quantitative variables have been presented as median and interquartile ranges and categorical variables as numbers and percentages. Assessment of normality and equality of variances was done with the Shapiro-Wilk test, and the Levene test, respectively. Continuous variables were compared with a Students' *T*-test and Mann-Whitney U test when appropriate. Pearson's correlation coefficient was used to assess the potential association between quantitative variables. Categorical variables were compared with Pearson's chi-squared test and Fisher's exact test.

Exact logistic regression analysis for small samples was used to identify predictors of outcome. Variables considered clinically relevant, and those statistically significant in the univariable analysis were included in the multivariable regression analysis.

Time-to-event analysis was performed by Cox survival modeling. Cox proportional hazards regression analysis was used to assess the influence of different variables on MACE. Kaplan-Meier curves for time to MACE were generated.

In addition, the area under the receiver-operator characteristic (ROC) curve was used to measure the discriminatory capacity of EAT and to find the cut-off point with the best sensitivity and specificity.

All tests were two-sided, and differences were considered statistically significant at *p-*values < 0.05. Statistical analyses were performed with Stata, version 14.1 (Stata Corp, Lakeway Dr. College Station, TX, USA).

This study was approved by our local Ethics Committee (CEIM). All procedures performed in the study were under the ethical standards of the institutional research committee and with the Declaration of Helsinki on human research. All patients gave written informed consent for the procedures performed in the study.

## Results

The median age of our study population (*n* = 41) was 55.8 [51.5–62.4] years. Of the total number of patients, 38 (93%) were male. From the total cohort, 37% of patients were hypertensive and 22% were diabetic, 39% were active smokers at the time of infarction and 22% were former smokers.

PPCI was performed in all cases. The median door-to-balloon time was 47.5 min [30.0–102.5]. The most frequently involved artery was the left anterior descending artery (ADA), which was responsible for AMI in 63.4% of cases. Twenty six patients (63%) had one-vessel disease, 9 patients (22%) had two-vessel disease and 6 patients (15%) had three-vessel disease. Regarding Gal-3 levels, the median was 16.5 ng/mL [12.7–25.2] at hospital admission.

Median LVEF was 58.4% [55.3–62.7]; EAT thickness at parasternal long axis view, was 4.1 [3.6–4.9] mm. Thirty five patients (85%) had LGE; the medians of infarct size were 15.3 g [8.2–24.6] and 12.4% [7.5–27.8]. Eleven patients (27%) showed MVO. Patients who had MVO had a larger thickness of epicardial fat compared to those without MVO (4.2 mm [3.7–5.3] vs. 3.7 mm [3.3–4.4], *p* = 0.050).

Patients with three-vessel coronary disease showed higher values of EAT thickness 5.0mm [3.7–6.6; *p* = 0.061]; these patients had worse LVEF (54.5% [46.1–62.7], 57.5% [56.4–62.1] and 58.4% [58.1–62.8] respectively, *p* = 0.0568) and GLS (−17.5% [−19.6–−14.6], −18.8% [−18.8–20.2] and −19.2% [−21.1–16.8] respectively, *p* = 0.078) compared to patients with one- and two-vessel disease.

The best cut-off value of EAT for MACE was 4.4 mm and had an area under the ROC curve of 0.767 (95% confidence interval 0.609–0.884), with an 80% sensitivity, 72.2% specificity, 28.6% positive predictive value, and 96.3% negative predictive value. After obtaining the cut-off value, patients were divided into two groups: EAT thickness <4 mm and EAT thickness ≥4 mm. Table 1 depicts the baseline characteristics of both groups.

**Table 1:** Baseline clinical characteristics.

	**EAT < 4 mm**	**EAT > 4 mm**	***p***
	**(*n =* 18)**	**(*n =* 23)**	
Age (years)	56.4 (47.8–63.8)	55.6 (51.5–62.4)	0.916
Male (n)	16	22	0.409
Weight (kg)	81 (70–92)	89 (78–95)	0.236
BMI	28.7 (24.2–30.4)	29.9 (26.3–31.5)	0.438
BSA	1.9 (1.8–2.1)	2.0 (1.9–2.1)	0.231
HTN (%)	27.8	43.5	0.300
DM (%)	11.1	30.4	0.138
DLP (%)	27.7	43.4	0.346
Smoking (%)	50.0	30.4	0.076
LDA (%)	70.6	56.5	0.509
RCA (%)	11.8	30.4	0.218
CX (%)	17.7	13.1	0.890
Multivessel CAD (n)	7	8	0.786
Killip I (%)	94	96	0.859
GRACE score	107 (95–122)	108 (89–118)	0.843
Time D-B (min)	54.6	79.1	0.235
Peak TnI	47.3 (8.3–56.6)	37.1 (9.4–120)	0.608
Peak CK-MB	62.8 (30.2–156.0)	161.3 (21.7–271.4)	0.281
TC (mg/dL)	182 (167–213)	174 (165–207)	0.392
HDL (mg/dL)	40.5 (33–43)	39 (34–48)	0.350
LDL (mg/dL)	120 (92–132)	103 (93–129)	0.622
TGC (mg/dL)	163.5 (97–254)	119 (90–164)	0.193
Cr (mg/dL)	0.91 (0.77–1.1)	0.90 (0.73–1.0)	0.572
GFr (ml/min)	83.7 (73.3–100.7)	87.8 (75.8–97.7)	0.789
Galectin-3 (ng/ml)	13.7 (12.2–21.3)	20.2 (13.7–29.1)	**0.027**

Data expressed as median and 25th−75th percentiles; number or percentage of patients. Bold values are statistically significant. BMI, body mass index; BSA, body surface area; CAD, coronary artery disease; Cr, creatinine; CX, circumflex coronary artery; D-B, door-to-balloon; DM, diabetes mellitus; DLP, dyslipidemia; GFR, glomerular filtration rate; HDL high-density lipoprotein; HTN, hypertension; LDA, left anterior descending artery; LDL low-density lipoprotein; RCA, right coronary artery; TC, total cholesterol; TGC, triglycerides.

In Table 2, baseline imaging characteristics of patients are shown. Left ventricular ejection fraction (LVEF) was significantly lower in patients with EAT >4 mm (55.7% [47.1–62.1] vs. 61.3% [57.0–63.2], *p* = 0.046) GLS was also significantly lower in this group, (−17.1% [−21.8-−15.7] vs. −19.4% [−22.4-−16.8], *p* = 0.042). E/e ratio was slightly higher in EAT >4 mm patients, although not statistically significant (10.1 vs. 8.6, *p* = 0.173). Left ventricular end-diastolic and systolic volumes, as well as left atrial volume index, were significantly larger at baseline in patients with EAT >4 mm. No other significant differences regarding TTE parameters between groups were found.

**Table 2:** TTE and CMR baseline parameters.

	**EAT < 4 mm**	**EAT > 4 mm**	***p***
	**(*n =* 18)**	**(*n =* 23)**	
IVS (mm)	12 (11–13)	11 (11–12)	0.554
PW (mm)	11 (10–11)	11 (10–11)	0.418
LVEDD (mm)	47 (42–50)	49 (47–52)	0.230
LVESD (mm)	34 (28–37)	35 (29–38)	0.654
LVEDV (ml/m2)	43.1 (38.9–50.5)	52.7 (45.6–61.2)	**0.005**
LVESV (ml/m2)	16.6 (14.5–20.5)	23.4 (19.4–28.2)	**0.001**
LV mass (g)	99 (89–111)	98 (90–114)	0.813
LVEF (%)	61.3 (57.0–63.2)	55.7 (47.1–62.1)	**0.046**
LV GLS (%)	−19.4% (−22.4–−16.8)	−17.1% (−21.8–−15.7)	**0.042**
LAVI (ml/m2)	23.6 (18.8–25.8)	27.8 (22.1–31.1)	0**.020**
E/A ratio	0.83 (0.62–1.2)	0.87 (0.71–1.3)	0.331
E/e' ratio	8.6 (7.5–9.6)	10.1 (7.1–11.1)	0.221
TAPSE (mm)	21.5 (19–23)	21 (19–25)	0.802
RV S' (cm/s)	11.8 (10.7–13.6)	12.4 (10.7–13.9)	0.674
PASP (mmHg)	25 (24–30)	21 (21–27)	0.349
LGE+ n (%)	13 (72)	23 (100)	**0.008**
MVO n (%)	5 (28)	6 (26)	0.714
IS (g)	9.6 (8.1–15.7)	19.2 (14.7–27.7)	**0.034**
IS (%)	7.9 (4.9–19.9)	21.1 (11.1–30.3)	**0.019**
ECV mean (%)	44.2 (35.8–57.3)	49.2 (40.8–58.7)	0.337
ECV infarct (%)	55.2 (36.7–74.5)	61 (43.3–86.6)	0.270
ECV remote (%)	18.5 (12.5–24.8)	25.4 (19.9–30.2)	0.071
Native T1 mean (ms)	1,132.4 (1,051.6–1,252.1)	1,269.7 (1,177.1–1,307.6)	**0.039**
T1 infarct (ms)	1,213 (1,116.9–1,315.5)	1,255 (1,111.5–1,364.6)	0.587
T1 remote (ms)	962.7 (893.9–992.9)	986.2 (938.1–1,033.6)	0.604

Data expressed as median and 25th−75th percentiles; number or percentage of patients. Bold values are statistically significant. ECV, extracellular volume; EDD, end-diastolic diameter; EF, ejection fraction; ESD, end-systolic diameter; EDV, end-diastolic volume; ESV, end-systolic volume; IS, infarct size; IVS, interventricular septum; LAVI, left atrial volume index; LGE, late gadolinium enhancement; LV, left ventricle; MVO, microvascular obstruction; PASP, pulmonary artery systolic pressure; PW, posterior wall; RV, right ventricle.

Regarding CMR, it was performed 5 days (3–7) after PPCI. All patients with EAT >4mm had LGE, compared to 13 (72%) in the other group (*p* = 0.008). In patients with EAT >4 mm, MVO was associated with lower LVEF at baseline 45.5% [40.0–46.1] vs. 56.3% [54.2–63.1], *p* = 0.027; and at 1-year follow-up, 46.7% [44.0–47.4] vs. 65.0 [60.2–71.0], *p* = 0.021.

LV infarct size was significantly larger in patients with EAT > 4 mm (19.2 g [14.7–27.7] vs 9.6 g [8.1–15.7], *p* = 0.021). Mean native T1 time was longer as well in these patients (1269 ms [1,177.1–1,307.6] vs. 1,132 ms [1,051.6–1,252.1], *p* = 0.039). In addition, we found a statistically significant correlation of baseline native T1 values with parameters of LV systolic function; negative for LVEF (r = −0.442, *p* = 0.007); and positive for GLS (r = 0.438, *p* = 0.006) and mean ECV (r = 0.551, *p* = 0.014). Baseline native T1 value also had a significant correlation with LVEDV (r = 0.666, *p* = < 0.001) and LVESV (r = 0.718, *p* = < 0.001) obtained at follow-up.

Gal-3 levels was significantly higher in patients with EAT >4 mm, (20.2 ng/ml (13.7–29.1) vs. 13.7 (12.2–21.3), *p* = 0.027). It is particularly noticeable that Gal-3 showed higher levels in patients with a BMI > 25 kg/m2 (17.7 [14.4–28.2] vs. 11.7 [7.8–13.1], *p* = 0.001).

At 1-year follow-up, patients with EAT >4 mm continued to have significantly higher LVEDV, (53.0 ml/m2 [46.3–60.9] vs. 44.9 ml/m2 [44.5–48.5], *p* = 0.021); as well as LVESV (21.1 ml/m2 [17.5–25.5] vs. 16.9 ml/m2 [17.5–25.5], *p* = 0.018). LVEF was lower in EAT >4 mm patients (60.2% [50.8–63.4] vs. 62.9 [56.3–68.0], *p* = 0.241); this pattern was also observed in LV GLS (−18.8% [−21.8–−14.3] vs. −20.1% [−21.1–19.0], *p* = 0.312). Regarding CMR data, native T1 mean time was significantly longer in the EAT >4 mm group (1,207.3 [1,138.4–1,267.5] vs. 1,124.6 [1,081.1–1,151.6], *p* = 0.041); and, interestingly, ECV was found to be higher in these patients, with a trend toward signification (44.5 [43.0–59.1] vs. 39.6 [29.3–44.3], *p* = 0.055). No statistically significant differences were found in other TTE or CMR parameters between groups.

As for Gal-3, it is worth mentioning that serum levels continued to be significantly higher in patients with EAT >4 mm at 1-year follow-up; 19.8 ng/ml [16.0–28.2] vs. 9.5 ng/ml [7.0–20.0], *p* = 0.031. Higher levels of Gal-3 observed in patients with BMI >25 kg/m2 at baseline were maintained at 1-year follow-up with a trend toward significance (19.8 [16.0–28.2] vs. 9.5 [7.0–19.9], *p* = 0.068).

### Major cardiovascular events during follow-up

Patients were followed up for a mean of 61 ± 13 months after the MI. In terms of events, after 1 year of follow-up, there were no MACE registered in any of both groups. At 5-year follow-up, in the group of EAT <4 mm, no events were registered. On the contrary, in the group of EAT >4 mm, 5 patients (22%) had MACE. Three patients were admitted for MI, 1 patient was admitted for heart failure and 1 patient died of non-cardiovascular causes.

There was no statistically significant association between the responsible coronary artery and the MACE (*p* = 0.562). Fifteen patients (66%) with EAT > 4 mm had one-vessel disease; four patients (17%) had two vessels disease and 4 patients (17%) had three-vessels disease (*p* = 0.672). No significant association between multivessel CAD and MACE was found (*p* = 0.246). It is worth mentioning that the number of affected coronary vessels did not show a significant association with LV systolic function parameters, either on admission or at follow-up; neither with the presence of LGE (*p* = 0.964) nor with infarct size (*p* = 0.456). Door-to-balloon time was longer in patients with EAT > 4 mm (47.5 min [30–105] vs. 40.0 min [20–86], *p* = 0.221) and consequently in those with MACE, although without statistical significance in any case.

Multivariable logistic regression analysis showed that only baseline TTE/CMR LVEF and EAT thickness were independent predictors of MACE (Table 3).

**Table 3:** Univariable and multivariable logistic regression analyses.

	**Univariable analysis**	***P* value**	**Multivariable analysis**	***P* value**
	**Odds ratio (95% CI)**		**Odds ratio (95% CI)**	
LVEDV	1.02 (1.01–1.05)	**0.046**		
LVESV	1.03 (1.00–1.07)	**0.040**		
LVEF	0.88 (0.78–0.99)	**0.036**	0.85 (0.75–0.97)	**0.047**
LV GLS	1.27 (0.94–1.64)	0.072		
LAVI	1.08 (0.93–1.26)	0.310		
E/e' ratio	1.03 (0.67–1.62)	0.866		
TAPSE	1.10 (0.87–1.39)	0.423		
PASP	0.88 (0.69–1.12)	0.300		
EAT	1.70 (1.03–3.56)	**0.015**	1.61 (1.01–4.10)	**0.041**
LGE	1.03 (0.99–1.06)	0.106		
MVO	2.25 (0.23–15.90)	0.416		
IS g	1.13 (0.99–1.28)	0.061		
ECV mean	0.95 (0.87–1.04)	0.276		
Native T1 mean	1.00 (0.99–1.01)	0.188		

Data are expressed as odds ratios (OR) and 95% confidence intervals (CI). EAT, epicardial adipose tissue; ECV, extracellular volume; EF, ejection fraction; IS, infarct size; LAVI, left atrial volume index; LGE, late gadolinium enhancement; LV, left ventricle; LVEF, left ventricular ejection fraction; LVEDV, left ventricular end-diastolic volume; LVESV: left ventricular end-systolic volume; LV GLS left ventricle global longitudinal strain. MVO, microvascular obstruction; PASP, pulmonary artery systolic pressure.

Bold values are statistically significant.

**Figure 2:**
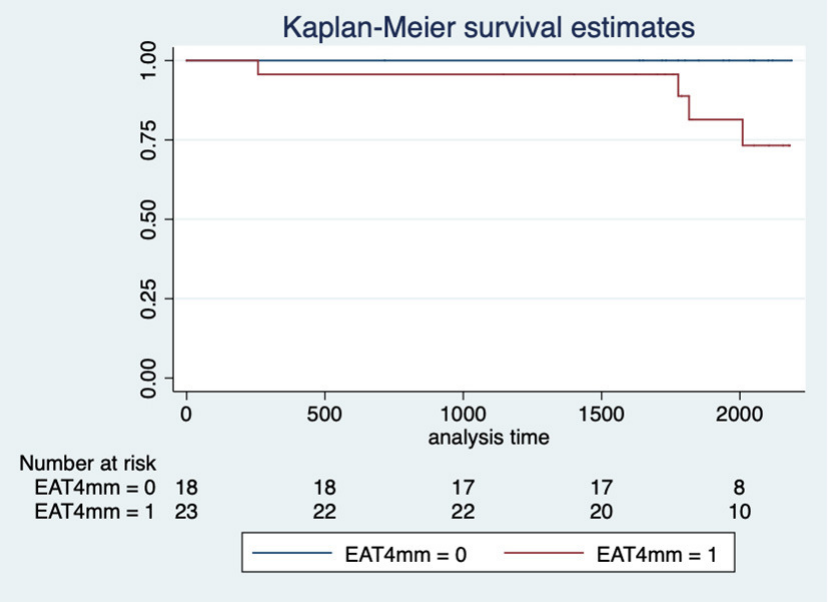
Kaplan-Meier curve of MACE after a MI by the thickness of EAT. EAT, Epicardial Adipose Tissue; MI, Myocardial Infarction.

By Cox proportional hazards regression analysis, the following variables were associated with MACE: LVEF, LVESV, LV GLS (both by CMR and echo), infarct size, and EAT (Table 4). Figure 2 shows the impact of EAT > 4 mm on the incidence of MACE.

**Table 4:** Cox regression analysis for MACE.

	**Univariable analyses HR (95% CI)**	***P*** ** Value**	**Multivariable analyses HR (95% CI)**	***P*** ** Value**
LVEDV	1.04 (1.01–1.07)	**0.023**		
LVESV	1.06 (1.02–1.11)	**0.007**		
LVEF	0.85 (0.75–0.97)	**0.013**	0.84 (0.73–0.97)	**0.019**
LVESV	1.10 (1.01–1.26)	**0.045**		
LV GLS	1.35 (1.08–1.69)	**0.008**		
EAT	3.50 (1.15–10.67)	**0.028**	5.59 (1.02–31.83)	**0.050**
Infarct size	1.18 (1.00–1.38)	**0.044**		

Data expressed as hazard ratios (HR) and 95% confidence intervals (CI). EAT, epicardial adipose tissue; LVEF, left ventricular ejection fraction; LVEDV, left ventricular end-diastolic volume; LVESV: left ventricular end-systolic volume; LV GLS left ventricle global longitudinal strain. Bold values are statistically significant.

## Discussion

The main results of our study are as follows: 1) patients with EAT >4 mm have worse LVEF and GLS, larger infarct size, and longer T1 values after MI; 2) higher levels of Gal-3 were observed in patients with EAT >4 mm; 3) a significant correlation between EAT >4 mm and native T1 values with LV systolic function and volumes was found, and 4) EAT >4 mm, along with LVEF, were independent predictors of MACE at 5-years follow-up.

EAT can be detrimental to the adjacent myocardium due to the release of pro-inflammatory and pro-fibrotic cytokines. It may play a role in the development and progression of CAD (2). The increase in EAT that is observed in overweight and obesity, impacts in different ways and contributes to vascular dysfunction and the development of cardiovascular disease (8). Ischemic insults, such as MI, could cause the activation of EAT to inflammatory signals in adipose stores adjacent to the myocardium, resulting in the magnification of vascular inflammation and plaque instability (9).

Quantification of EAT and its relation with cardiovascular disease has been described in several publications (2–5); in our study, the best cut-off value to predict outcome was 4 mm. Previous studies have linked EAT to CAD; Ahn et al. (10) described that epicardial fat thickness values > 3.0 mm were independently associated with the presence of CAD. It has been described that epicardial fat thickness tends to be higher in patients with CAD and unstable angina than in subjects without CAD and or stable angina. Moreover, EAT seems to have an impact on the development and progression of CAD regardless of the presence of other risk factors and the distribution of fat depots. On the other hand, it seems that the location of this adipose tissue is fundamental in the development of CAD; according to some authors, pericoronary adipose tissue (PCAT) is associated with the severity of CAD through an imbalance between harmful and protective adipocytokines released (10–13).

The presence of PCAT represents a specialized local depot that in the context of obesity and insulin resistance, oxidative stress, and inflammation, promotes a rise in pro-inflammatory cytokines, including tumor necrosis factor-alpha and interleukins. The complex interaction of pro-inflammatory cytokines with endothelial cells produces reactive oxygen species, vascular cell adhesion molecules, and E-selectin. These substances induce a complex biochemical response whose final step is to stimulate the conversion of fibroblasts into myofibroblasts, which deposit collagen and thus produce myocardial fibrosis (14–16). A previous study in an animal model showed that EAT contains a high density of lymphoid clusters, which expands in response to AMI; this inflammatory reaction induced by EAT produces, by different pathways, myocardial fibrosis and consequently LV dysfunction (16).

Our research showed high levels of serum Gal-3 in patients with EAT >4 mm. Gal-3 is a pro-inflammatory cytokine that is by activated macrophages. Gal-3 is known to play a role in the inflammatory, fibrosis, and scarring processes associated with cardiac remodeling that occur in heart failure and AMI (17). It has been described, as an independent risk factor for MACE in ACS patients, for every 1 ng/ml increase in Gal-3 levels, an elevation of MACE rate is observed (18).

Previously, our group reported that high levels of Gal-3 are associated with diastolic dysfunction in obese patients. Moreover, the inhibition of Gal-3 activity in an experimental model was associated with a decrease in myocardial fibrosis and inflammation at cardiac and vascular levels (19).

Myocardial fibrosis is, apparently, an important determinant in the evolution of patients with MI and, CMR, using native T1 mapping, represents a non-invasive assessment of diffuse myocardial fibrosis and correlates with subclinical myocardial dysfunction and the extent of ventricular remodeling in diverse clinical scenarios (20).

Findings of Toya et al., described lower LVEF, larger LV infarct size, and more prevalent MVO, and found an independent association between worse MACE-free survival in STEMI patients and higher amounts of perivascular EAT (21).

These results are consistent with the findings of our study. Patients with EAT >4mm had larger infarct size and longer native T1 values; both parameters were associated with the presence of lower LVEF and GLS in these patients. In the study published by Marques et al. (22), native T1 times were associated with incident mortality and described a higher unadjusted cumulative survival probability in patients with native T1 <954 ms. Higher values of native T1 were associated with worse cardiovascular outcome and increased mortality rates. This study also showed that for every 10 ms increase in native T1, there was a higher hazard ratio for the prediction of mortality and MACE (22). T1 mapping has demonstrated the capability to accurately determine the amount of diffuse myocardial fibrosis; this diffuse fibrosis and changes in the ECV are more associated with poor outcome than focal fibrosis in different clinical scenarios (23–25). Also, some studies have shown that the detection of diffuse interstitial myocardial fibrosis has been associated with higher mortality in diverse cardiac diseases (26).

In this sense, there is evidence that suggests a relationship between ECV expansion and adverse outcomes in patients with heart failure. Particularly, in animal models of diastolic dysfunction and patients with heart failure and preserved LVEF, the severity of myocardial fibrosis proved to be a predictor of mortality and MACE (27, 28). Wong et al., have described the significant relationship between quantitative ECV measures and mortality; expansion of ECV could indicate the replacement of healthy myocardium by damaged and dysfunctional myocardium (29).

Other CMR-derived parameters, such as the myocardial area at risk, the myocardial salvage index, and the MVO have been described (30); despite larger amounts of epicardial fat in patients who had MVO, we did not observe an association between MVO and MACE during follow-up.

Noteworthy, in our cohort, patients with EAT >4 mm, showed a higher ECV at the CMR performed 1 year after the AMI; this might be mainly related to the inflammatory response caused by myocardial ischemia which conduces to cardiomyocyte apoptosis and further structural changes, such as ECV expansion and myocardial fibrosis, which in turn leads to LV enlargement and systolic function worsening, as observed in these patients.

As mentioned, the potential structural changes in LV geometry observed in patients with EAT >4 mm could be related to the chronic inflammatory process and the associated fibrosis. Parisi et al. reported an association between changes in the thickness of EAT and reduced LVEF, regardless of the infarct size; this supports the role of inflammation in ventricular function and remodeling (31). In their study, it is described that EAT thickness was associated with ventricular remodeling; specifically, a positive increase in EAT was closely related to larger LV volumes (31).

Our results show that EAT thickness >4 mm was associated with larger LVEDV and LVESV, as well as with worse systolic function parameters, both at baseline and follow-up. There is a reasonable amount of information to support that immune response and inflammation processes have a pivotal stake in the onset and development of LV remodeling and dysfunction; is therefore possible to assume the hypothesis that EAT, because of its pro-inflammatory influence, may contribute to LV remodeling and dysfunction in the context of CAD and myocardial ischemia, through a complex pathophysiologic process of myocardial fibrosis (32, 33).

Finally, it is especially noteworthy the low rate of MACE observed in our cohort; there is nothing new under the sun that mortality after having an AMI has declined progressively; early intervention and noticeable improvement in pharmacological treatment, as well as, risk factors control, play a fundamental role in this regard (34). Infarction code protocol was launched in Spain in 2000 with full national coverage achieved in 2017. In Spain, mortality rates, both in-hospital and 30-day have significantly decreased since the implementation of the program “Infarction Code Networks” (35). This is reflected in our cohort, in which no MACE occurred during the first year of follow-up; nevertheless, after a 5-years follow-up, we observed that patients with EAT > 4 mm have a worse systolic function in terms of LVEF and GLS, LV remodeling with larger end-diastolic and end-systolic volumes, all of which are related to the occurrence of MACE.

Given all the above, and the large amount of information that exists in this regard, it is reasonable to emphasize that EAT might be considered in the mid and long-term risk assessment of patients with a first AMI and possibly also in the general population due to its involvement in the development and progression of various cardiovascular diseases (36, 37).

### Limitations

This is a single-center, observational study with both the inherent limitations of this type of design. The sample size is small, and all patients were treated with percutaneous coronary intervention; therefore, the representativeness of the sample may not accurately reflect the characteristics of the general population suffering a first MI. Inflammation markers, such as Gal-3, as well as CMR sequences for T1 mapping or ECV quantification, are not included in MI diagnosis and treatment protocols in the corresponding clinical practice guidelines, so larger studies are needed to confirm the role of these markers and imaging parameters in the diagnosis, treatment, and prognosis of MI.

## Conclusions

EAT thickness is a feasible, non-invasive, low-cost parameter that might provide crucial information on the chronic inflammatory process after an AMI. Patients with EAT > 4 mm had worse systolic function parameters, larger infarct size and longer T1 values after a MI, as well as a higher incidence of MACE at 5-year follow-up. The multi-modality imaging approach to patients with a first AMI allows tissue and functional characterization that has important prognostic implications for left ventricular systolic function and patient outcomes.

## Data availability statement

The raw data supporting the conclusions of this article will be made available by the authors, without undue reservation.

## Ethics statement

The studies involving human participants were reviewed and approved by CEIM–Hospital Clínico San Carlos. The patients/participants provided their written informed consent to participate in this study.

## Author contributions

FI and EG collected data and wrote the article. FI and CO performed the statistical analysis. VC, GM, and EM-M performed all blood test analyses. All authors reviewed the article and sent their contributions. All authors have seen and approved the submitted manuscript, have contributed significantly to work, attest to the validity and legitimacy of the data and its interpretation, and agree to its submission to Frontiers in Cardiovascular Medicine.

## Funding

Funded by Instituto de Salud Carlos III-Fondo Europeo de Desarrollo Regional (FEDER) (PI18/00257, PI21/00431, CIBERCV, Madrid, Spain).

## Conflict of interest

The authors declare that the research was conducted in the absence of any commercial or financial relationships that could be construed as a potential conflict of interest.

## Publisher's note

All claims expressed in this article are solely those of the authors and do not necessarily represent those of their affiliated organizations, or those of the publisher, the editors and the reviewers. Any product that may be evaluated in this article, or claim that may be made by its manufacturer, is not guaranteed or endorsed by the publisher.
